# A Mixed-Method Study to Determine the Benefits of Periconceptional Folic Acid Supplementation and Effects of Folic Acid Deficiency in Mothers on Birth Outcomes

**DOI:** 10.2196/resprot.5722

**Published:** 2016-06-23

**Authors:** Gudlavalleti Venkata S Murthy, Sunanda Reddy Kolli, Sutapa B Neogi, Samiksha Singh, Komal Preet Allagh, Neena John, Srinivas N., Sudha Ramani, BR Shamanna, Pat Doyle, Sanjay Kinra, Andy Ness, Dinesh Raj Pallepogula, Hira B Pant, Smiksha Babbar, Raghunath Reddy, Rachna Singh

**Affiliations:** ^1^ Indian Institute of Public Health Hyderabad India; ^2^ Centre for Applied Research and Education on Neurodevelopmental impairments and Disability-related health initiatives (CARENIDHI) New delhi India; ^3^ Indian Institute of Public Health, Delhi Delhi India; ^4^ Indian Institute of Public Health, Bhubaneswar Bhubaneswar India; ^5^ Indian Institute of Public Health- Bangalore Bangalore India; ^6^ University of Hyderabad School of Medical Sciences Hyderabad India; ^7^ London School of Hygiene and Tropical Medicine London United Kingdom; ^8^ London School of Hygiene & Tropical Medicine London United Kingdom; ^9^ University of Bristol Bristol United Kingdom; ^10^ Indian Institute of Public Health Delhi India

**Keywords:** folic acid, neural tube defects, orofacial clefts, periconceptional folic supplement, key informant method

## Abstract

**Background:**

Evidence from high income countries shows mothers who are supplemented with folic acid in their periconceptional period and early pregnancy have significantly reduced adverse outcomes like birth defects. However, in India there is a paucity of data on association of birth defects and folic acid supplementation. We identified a few important questions to be answered using separate scientific methods and then planned to triangulate the information.

**Objective:**

In this paper, we describe the protocol of our study that aims to determine the association of folic acid and pregnancy outcomes like neural tube defects (NTDs) and orofacial clefts (OFCs). We decided to fill the gaps in knowledge from India to determine public health consequences of folic acid deficiency and factors influencing dietary and periconceptional consumption of folic acid.

**Methods:**

The proposed study will be carried out in five stages and will examine the questions related to folic acid deficiency across selected locations in South and North India. The study will be carried out over a period of 4 years through the hierarchical evidence-based approach. At first a systematic review was conducted to pool the current birth prevalence of NTDs and orofacial clefts OFCs in India. To investigate the population prevalence, we plan to use the key informant method to determine prevalence of NTDs and OFCs. To determine the normal serum estimates of folic acid, iron, and vitamin B12 among Indian women (15-35 years), we will conduct a population-based, cross-sectional study. We will further strengthen the evidence of association between OFCs and folic acid by conducting a hospital-based, case-control study across three locations of India. Lastly, using qualitative methods we will understand community and health workers perspective on factors that decide the intake of folic acid supplements.

**Results:**

This study will provide evidence on the community prevalence of birth defects and prevalence folic acid and vitamin B12 deficiency in the community. The case-control study will help understand the association of folic acid deficiency with OFCs.

**Conclusions:**

The results from this study are intended to strengthen the evidence base in childhood disability for planning and policy initiatives.

## Introduction

### Background

Folic acid is a micronutrient with a vital role during human reproduction and in a variety of physiological processes [1,2]. A report from the World Health Organization [3] indicated that the percentage of pregnant women with a serum folic acid level less than 3 ng/mL was highest among pregnant women in Sri Lanka (57%), followed by India (41.6%). Poor serum folic acid levels are linked with negative health outcomes in pregnancy like abruptio placentae, preeclampsia, spontaneous abortion, congenital heart defects, stillbirth, preterm delivery, low birth weight, and serious birth defects of the brain and spine [4]. Studies from developed countries have shown folic acid deficiency is strongly associated with birth defects, particularly neural tube defects (NTDs) and orofacial clefts (OFCs) [1,5]. Evidence from some countries shows that mothers who are supplemented with folic acid in their periconceptional period and early pregnancy have significantly reduced adverse outcomes compared with mothers who are not similarly supplemented [6].

The review of the 4^th^ Millennium development goal (reduce child mortality) shows that the global under-5 child mortality has drastically dropped by more than half [7]. In India, the current under-5 child mortality is estimated to be 48 per 1000 live births, a little over the set target [8]. However, this decline is attributed to a fall in the number of deaths from infectious diseases and malnutrition; while the mortality attributed to birth defects remains constant [9]. The most common birth defects in India are NTDs [8]. OFCs are also an important birth defect in India.

There are currently no large studies from India that report the normal folate levels among Indian women and periconceptional dietary consumption or supplementation of folic acid. The periconceptional folic acid supplementation is challenging to incorporate in the national programs. In the absence of baseline evidence, planning and monitoring will be difficult. There are no large studies on knowledge of folic acid and its importance among laypersons in India. Only one small study demonstrates that the awareness is poor, 10 of 50 women knew of folic acid, and none of them linked it with birth defects [10]. Studies have also shown that awareness among health providers about periconceptional supplements is inadequate [11,12], while many physicians were generally aware of the linkage between folic acid and birth defects, their knowledge of timing and dosage was found to be lacking.

India also does not have evidence for population prevalence of NTDs or OFCs and association of low folic acid levels and birth defects. India is one of the many countries of the world where population estimates of the prevalence of birth defects are not routinely collected [13]. Currently, there is no national registry for birth defects. Hospital-based surveys or studies are the most common source of information on birth defects like NTDs and OFCs in India. These studies have reported inconsistent results. We conducted a systematic review to determine the current birth prevalence of NTDs and OFCs in India [14]. Our review revealed that there was just one population-based study reporting prevalence of NTDs from a rural district of North India [15]. There have been no population-based, high-quality studies reporting prevalence of NTDs and OFCs from South India. The review also pointed out significant regional differences in pooled prevalence of both NTDs and OFCs [14].

The only population-based prevalence study from North India used the door-to-door survey method to identify cases of NTDs [15]. A door-to-door survey is time consuming and expensive [16]. The key informant method (KIM) [17] may be a faster and more cost-effective method to estimate population prevalence’s. KIM has been successfully used in the past in identifying children with disabilities in various low- and middle-income countries like Bangladesh, China, Ethiopia, Iran, Malawi, and Nigeria [18-26]. In India, only a few studies have considered Integrated Child Development Service (Anganwadi) workers and other community-based personnel as key informants (KIs) to identify childhood disability [27,28].

We decided to fill the gaps in knowledge from India to determine public health consequences of folic acid deficiency and factors influencing dietary and periconceptional consumption of folic acid. We identified a few important questions to be answered using separate scientific methods and then we plan to triangulate the information. To investigate the population prevalence and to capture regional differences, we plan to carry out a population-based prevalence study of birth defects (NTDs and OFCs) in a rural community of South India and urban community from North India using KIM. As a part of the prevalence study, we will elicit history of specific dietary practices and folic acid intake during the affected pregnancy. This will provide evidence on association between dietary folic acid and birth defects. We will also conduct a population-based, cross-sectional study among women in the reproductive age group (15-35 years) in South India, to determine normal serum estimates of folic acid, iron, and vitamin B12.

A review of literature on association of folic acid and OFCs shows there is some suggestive evidence for the role of folic acid in prevention of OFCs [29]. However, the observational case-control studies conducted so far have limitations of recall bias and biased report of use based on the pregnancy outcome of OFCs [29]. Yet, many studies have shown that knowledge about consuming folic acid periconceptionally among women is poor [30-34]. To build on the evidence gathered in our study, we will conduct a hospital-based, case-control study to look at the association between folic acid deficiency during pregnancy and OFCs. We are not including NTDs in the case-control study as the child survival until 4 months of age is very low [35]. In order to prevent recall bias, we will be eliciting history of folic acid intake and dietary practices among cases within 4 months of birth and from controls within 48 hours of birth. One case-control study has been previously conducted in India, and shows no significant association between the serum folate levels of mothers of children with or without clefts [36]. However, in this study, cases were included until the age of 14 months, by which time the maternal folate levels would have fluctuated as well as recall bias would have set in. The sample size of this study was very small with just 28 cases of nonsyndromic clefts.

In order to triangulate the information, we will conduct a qualitative study to determine the community and the health provider's perspective to the factors that decide the intake of folic acid supplements, and the health-seeking behavior among pregnant women in the community.

### Study Aim

The aim of this study is to determine the public health consequences of folic acid deficiency among mothers in India. Also, to study the association of folic acid deficiency with pregnancy outcomes especially NTDs and OFCs.

### Ethical Approval

The ethics committee of the Public Health Foundation of India (TRC-IEC-117/11) has granted favorable ethical approval for this study. Ethical approval was obtained in June 2012.

## Methods

### Design

The research proposed in five stages will examine the questions related to folic acid deficiency across selected locations in South and North India and its related consequences through the hierarchical evidence-based approach. The overall study duration will be 4 years. All data will be archived in an anonymized format at the end of the study and will be password protected to ensure that access is restricted to the investigators. Table 1 shows an overview of the five stages of this study with the expected outcome and analysis plan.

**Table 1:** Overview of study stages.

Stage	Study method	Study location	Tools used	Expected outcome	Analysis plan
Stage 1	Systematic review	India	Review manager	Birth prevalence of NTDs and OFCs in India	Meta-analysis using Review manager. Random and fixed- effect model to calculate pooled prevalence
Stage 2	Quantitative/ prevalence study	Mahbubnagar district, Telangana and North east Delhi	Flip book for training KIs^a^, KI profile, line listing format, and semistructured questionnaire for cases on: sociodemography, antenatal, intrapartum history, and life style behavior	Population prevalence of NTDs^b^, OFCs^c^, microcephaly, hydrocephalus, visual impairment, hearing impairment, motor impairments and intellectual impairments among children less than 6 years of age Prevalence of stillbirths in the community Descriptive statistics	Database will be developed in MS Access. STATA 14.0 will be used for analysis
Stage 3	Estimation of serum folic acid, ferritin and vitamin B12 levels	Mahbubnagar district, Telangana	Semistructured questionnaire on sociodemographic and reproductive history, and blood samples; anthropometric measurements	Normative values for serum folic acid, ferritin and vitamin B-12 among women in 15 to 35 years age group Body mass index of women in the age group 15 to 35 years Prevalence of folic acid and Vitamin B12 deficiency Hemoglobin estimation	Analysis of blood sample by solid phase radioimmunoassay method
Stage 4	Qualitative study	Mahbubnagar district, Telangana	Topic guide for focus group discussions, semistructured questionnaire for case studies, and interview schedule for in-depth interviews	Community perspectives on NTDs and OFCs Sociocultural factors that affect the uptake of folic acid supplements Health-seeking behavior during pregnancy Attitudes on periconceptional folic acid supplementation National Program and policy perspectives from experts	Computer assisted thematic analysis (Atlas-Ti software)
Stage 5	Case control study- hospital based	Hyderabad Bangalore and Delhi	Semistructured questionnaire on sociodemographics, reproductive, medical, occupational history and consumption of periconceptional folic acid supplements	Association between folic acid supplementation and OFCs	STATA 14.0 will be used for analysis, and calculation of odds ratio

^a^Key informants.

^b^Neural tube defects.

^c^Orofacial clefts.

### Stage 1: Systematic Review

At the outset, a systematic review was conducted to summarize the existing research evidence on the prevalence of NTDs and OFCs among live births and stillbirths in India using data from all available community- and hospital-based observational studies [14]. A comprehensive literature search for observational studies was conducted on MEDLINE and EMBASE databases using medical subject heading terms (neural tube defects OR cleft lip OR cleft palate AND prevalence AND India). We included all hospital- or community-based studies determining the birth prevalence of OFCs or NTDs. Nineteen studies met our inclusion criteria, with only one community-based study. Subgroup analysis was performed for region, time period, consanguinity, and gender of newborn.

Meta-analysis was performed using Review Manager software. We obtained a pooled birth prevalence of 4.5 per 1000 total births (95% confidence interval [CI] 4.2-4.9) for NTDs and 1.3 per 1000 total births (95% CI 1.1-1.5) for OFCs using the random-effect model. There was a significant variation in the prevalence of both NTD and OFCs across regions of India [14].

### Stage 2: Population Prevalence study (KIM)

#### Objectives

1. To estimate the prevalence of specific birth defects: NTDs, OFCs, microcephaly, hydrocephalus, visual impairment, hearing impairment, motor impairment (cerebral palsy, club foot, muscular dystrophy, poliomyelitis, phocomelia), and still births among children less than 6 years of age in the community using the KIs and health workers in Mahbubnagar district (Telangana) and Northeast Delhi (Delhi)

2. To determine the proportion of disability caused by OFCs and NTDs among all the specified birth defects in children less than 6 years in Mahbubnagar district (Telangana) and Northeast Delhi (Delhi)

3. To assess whether key informants can identify birth defects that are visible to the naked eye

4. To describe epidemiological characteristics (maternal characteristics such as age, parity, consumption of folic acid supplements, residence, etc) of specified birth defects in Mahbubnagar district (Telangana) and Northeast Delhi (Delhi).

#### Study Population

Children in the age group 0 to 6 years (born between 2008 and 2013), both live and stillbirths with any birth defect will be recruited in the study. The respondent will be the mother of the child. If she is not present (unavailable inspite of three visits or dead), the child will be included in prevalence but will not be recruited for eliciting the detailed history.

#### Sample Size and Study Sites

We used a combined prevalence of NTDs and OFCs of 10 per 10,000 births [37,38], 20% precision and alpha of 0.1 to estimate the sample size. We calculated a total sample size of 44,824 for the study. We plan to include two regions of India to conduct the community-based prevalence study. One site is in North India (Delhi) and the other in South India (Telangana).

Purposive sampling has been used to identify our study sites within the two states. In Telangana, we have included seven rural mandals (smallest administrative unit) of Mahbubnagar district (Addakal, Bhoothpur, Bijinapalle, Jadcherla, Ghanpur, Mahbubnagar (rural), and Thimmajipet). As per Census 2011, the total population of these seven mandals is 355,043 [39] and the population of children aged 0 to 6 years is 48,016 [40].

In Delhi, we have included seven urban regions from Northeast Delhi (Shahadra, Seemapuri, Seelampur, Babarpur, Gautam vihar, Wazirabad, and Anand mansarovar). The total population from these seven regions is 335,805 [39] and the population of children aged 0 to 6 years is 38,407 [41].

#### Study Design

The KIM [17] will be used to identify children (0-6 years) with birth defects in the community. The KIs will be Anganwadi workers, Accredited Social Health Activist, members of Disabled Peoples Organization, and self-help groups from within the study sites. We will recruit one KI for every 1000 population. The KIs will undergo a 1-day training on identification of specific birth defects among children: NTDs, OFCs, microcephaly, congenital hydrocephalus, visual impairment, hearing impairment, motor impairment (cerebral palsy, clubfoot, muscular dystrophy, poliomyelitis, and phocomelia), and intellectual impairment. Using a flipbook and a PowerPoint presentation, our research team will conduct the training. We are training the KIs on identifying birth defects other than NTDs and OFCs so that we can determine the proportion of children with NTDs and OFCs among all the visible birth defects in the community.

The flipbook consists of 60 images depicting the various birth defects mentioned above. The flipbook also consists of images depicting foods rich in folic acid, images of folic acid tablets and ways to prevent NTDs, OFCs, and other birth defects. From each administrative region (approximately 100,000 population), two to three batches of 20 to 22 KIs will be trained. The flipbook has been pretested in the community and changes incorporated in the images to make it culturally appropriate. The flipbook is for training purposes only and will not be given to KIs for use in the field. The pictures in the flipbook are thought to be disturbing if showed to the layman in the field. However, sketches of all the disabilities will be provided in a handout for their reference in the field.

The KIs will do line listing for the 1000 population in which she usually caters her community services too. The KIs will be given 2 weeks to complete listing of cases of birth defects and stillbirths from their specified population, after the training is completed. The completed line listing forms will be handed over to the field investigator, who will confirm and verify the addresses of all the children listed. After the listing is completed, a trained medical doctor will visit each of these children’s homes and examine them to confirm the diagnosis. Written informed consent will be obtained from the mother for examining the child. For all the confirmed cases of specified birth defects, the field investigator will document sociodemographic-, pregnancy-, and birth-related history and risk factors using a prestructured questionnaire from the mother of the affected child.

The mother of each child identified with birth defects will also be asked if she is aware of any other child with a similar birth defect in the vicinity. This will help in identifying any case, which may have been missed by the KI. In order to triangulate the data, a list of children with birth defects will be obtained from the Society for Elimination of Rural Poverty in Telangana and Smile Train Project. This will be used to compare and identify the missed cases if any.

Prevalence will be calculated as the number of cases of birth defect identified in the district using the KIM divided by the estimated number of children (0-6 years) alive in that district as per the Census data.

#### Statistical Analysis

A database will be developed in MS ACCESS. STATA 14.0 will be used for data analysis.

### Stage 3: Estimation of Serum Folic Acid, Ferritin, and Vitamin B-12

This study is being conducted in association with National Institute of Nutrition (NIN) at Hyderabad. It is part of a concurrent study at NIN looking at the nutritional status of women in the age group 15 to 35 years of age in Mahbubnagar district of Telangana.

#### Objective

1. To estimate normative values of serum folic acid, ferritin, and vitamin B12 among women in the age group 15 to 35 years in Mahbubnagar district, Telangana

2. To estimate the prevalence of folic acid and vitamin B-12 deficiency among 15 to 35 years women in the community (Mahbubnagar district, Telangana).

#### Sample Size and Study Site

For calculating the sample size, we used unpublished data from NIN on mean folic acid levels and standard error for women in the reproductive age group. Using the mean folic acid level of 10 ng/mL and standard error of 1.1, and a precision of 0.1 and 0.05 alpha, we calculated a sample size of 465. Thus, for this study, we will recruit 500 women in the reproductive age (15-35 years) from Mahbubnagar district, Telangana.

#### Study Design

This is a community-based, cross-sectional study. Women in the age group 15 to 35 years, irrespective of their marital, pregnancy, and lactation status, will be recruited in the study. Written informed consent will be obtained from the participants before registering and conducting any interview or blood examination. Any woman who is seriously ill or refuses to participate will be excluded from the study. A multistage, stratified, two-stage, random sampling method will be used. To get a representative sample, we will randomly select one mandal from each revenue division of the Mahbubnagar district. Thus, five mandals will be randomly selected. From each mandal, three villages will be randomly selected. Within each selected village, we will randomly select 30 to 35 households with women 15 to 35 years. From each household, only one woman will be randomly selected for the study.

The field investigator will administer a semistructured questionnaire eliciting sociodemographic and reproductive history and take anthropometric measurements. A trained lab technician will then draw 5 to 10 mL of blood from the participants under aseptic conditions. The blood sample will be centrifuged in the field, within 6 hours and stored at −20° C in a laboratory in the mandal. The samples will be transported to NIN, Hyderabad thrice a week for analysis.

At NIN, plasma levels of vitamin B12 and folic acid will be measured by solid phase radioimmunoassay method using a commercially available kit designed for simultaneous measurement of vitamin B12, ferritin and folic acid [42]. Hemoglobin estimation will be done. We will estimate the prevalence of folic acid and vitamin B12 deficiency among the women.

#### Statistical Analysis

The data will be analyzed using SPSS and a normogram will be plotted. The mean and median level of folic acid, ferritin, and vitamin B12 levels will be ascertained. Appropriate adjustments for potential confounder effects will be made.

### Stage 4: Community and Program Perspectives on NTDS, OFCs, and Periconceptional Folic Acid Supplementation

#### Objectives

1. To explore community perspectives among both men and women, on birth defects like NTDs and OFCs, using case studies and focus group discussions (FGDs) in Mahbubnagar district, Telangana

2. To examine sociocultural factors and their effect on uptake of folic acid supplementation during the periconceptional period by using interviews with health care staff in Mahbubnagar district, Telangana

3. To examine program perspectives on periconceptional folic acid supplementation by using in-depth interviews (IDIs) among personnel working in National program against anemia.

#### Study Design

This is a qualitative study. Data for this aspect of the study will be collected through a mix of qualitative data collection tools such as FGDs, IDIs, and case studies. In order to get a holistic picture of the knowledge and perceptions of NTDs and OFCs and the link of these with folic acid, data will be collected from the community as well the health system. Then the results will then be triangulated.

#### Study Site

The study will be conducted in the four Mandals of Mahbubnagar district of Telangana. These mandals have been purposively chosen to cover a diverse population that is depictive of the natural setting in this region: Bijinapalle (majority population is Scheduled caste and tribes), Jadcherla (majority tribal population), Mahbubnagar (predominantly Muslim population with easy access to health care), and Ghanpur (predominantly Muslim population with poor access to health care).

#### Sampling and Data Collection Methods

For objectives 1 and 2, data will be collected from the community using FGDs and case studies. Because some of the themes are gender-sensitive, FGDs will be conducted separately with men and women. The FGDs are intended to elicit community perceptions on local ideas of causation, prevention, and treatment of NTDs and OFCs. FGDs with women also include themes on practices during pregnancy and childbirth that might have repercussions on NTDs/OFCs.

The composition of persons participating in the FGDs will be purposively selected. The study population we cover includes a mix of scheduled castes, tribes, Muslim minorities, and persons who reside in the semiurban regions. We intend to conduct discussions with each of these four groups. An indicative number of FGDs in each of the mandals is given in Table 2. The principle of data saturation will be used to guide the actual number of FGDs conducted.

In addition to the FGDs with the community, we will be doing individual interviews (case studies) with families having affected children and with affected adults. Here, we chose to do interviews because we felt that responses to certain questions such as stigma, testing, and ways through which the disease affects their life, may be personal and people may be hesitant to share such ideas in a group. The participants for case studies will also be purposively chosen so as to elicit rich and illustrative perspectives on themes such as living with OFCs, coping with a child having NTDs/OFCs, stigma in different stages of live, gender differences in coping, and access to care. We intend to do approximately 10 to 15 case studies in the four mandals.

For objective 2, we will be collecting data through IDIs with health system staff including doctors, nurses, auxiliary nurse midwife (ANM), and other community level workers. These interviews will help us understand issues pertinent to the knowledge of NTDs/OFCs among health workers and issues with the supply of folic acid and its supplementation. Here, we choose the interview method over FGDs due to the logistic difficulties in getting health system staff together in one place. We will supplement this information with data from interviews with personnel working in the National program against anemia (objective 3). Overall, this set of interviews will help us envisage barriers to the supplementation of folic acid from the perspective of the health system; and the feasibility of a periconceptional folic acid program in India. All interviews will be conducted in the local language (Telugu and Deccani Hindi). Table 2 gives an indicative number of the number of interviews to be conducted within the health system; the concept of data saturation will be used to guide the actual numbers.

**Table 2:** Data collection for qualitative study (indicative numbers).

		In-depth interviews	In-depth interviews	Focus group discussions	Focus group discussions	Case Studies
**Type of population**											
		Health system	Experts and personnel in National anemia program	Community (General)	Community (women in reproductive age group)	Families with affected children/Adults with NTD^a^/OFC^b^
**Name of mandal**						
	Bijinapalle	Doctor -3 Nurse-3 ANM^c^/ASHA^d^- 6		Scheduled Tribe- 1 Scheduled Caste-2	Scheduled Tribe -1 Scheduled Caste -2	
	Jadcherla	Doctor -1 Nurse-1 ANM/ASHA- 2		Scheduled Tribe -2	Scheduled Tribe-2	
	Mahbubnagar	Doctor -1 Nurse-1 ANM/ASHA- 2		Muslim-1 Semiurban-2	Muslim-1 Semiurban-2	
	Ghanpur	Doctor -2 Nurse-2 ANM/ASHA- 4		Muslim-1	Muslim-1	
**Total**						
		Doctor -7 Nurse-7 ANM/ASHA- 14	As required (5-10)	8 FGDs	8 FGDs	To choose depending on number/ type of cases from the KI^e^ study –approximately 10-15

^a^Neural tube defects.

^b^Orofacial cleft.

^c^Auxiliary nurse midwife.

^d^Accredited social health activist.

^e^Key informant.

#### Data Analysis

Data analysis for this study will be initiated concurrently with the data collection process, so as to allow the team to refine the questions, develop hypothesis, and pursue emerging avenues of inquiry for IDIs subsequently. All the FGDs, case studies, and IDIs will be translated into English and transcribed. Generic thematic analysis techniques will be used for the study. Analyses will be initiated with a list of predefined themes and new themes will be incorporated as these emerge. For data reduction, the qualitative analysis software ATLAS-TI will be used.

### Stage 5: Case Control Study (Hospital-Based)

#### Objectives

The objective is to determine if mothers of children with OFCs are more likely to have folic acid deficiency during the periconceptional period and early pregnancy (first trimester) in hospital-based settings in Delhi, Hyderabad, and Bangalore. In order to prevent recall bias among the respondents, we will be eliciting history of folic acid intake and dietary practices among cases within 4 months of birth and from controls within 48 hours of birth. In this study, we did not include NTDs, as it was not logistically feasible to capture cases at the time of birth. If we increased the case population to less than 4-months old then due to high mortality of NTDs we will not get the required sample size.

#### Sample Size and Study Site

In terms of power and sample size, it is estimated with 90% power assumed to detect a 50% increase in risk (odds ratio=1.5) associated with a risk factor (periconceptional folic acid intake) present in the controls group at a prevalence of approximately 20% and assuming nonresponse rate of 5%. The required sample size would be 150 cases and 600 controls. Hence, this total sample size will be recruited cumulatively from three sites.

#### Study Design (Recruitment of Cases and Controls)

This is a hospital-based case control study that will be conducted simultaneously in three cities of India: Hyderabad, Delhi, and Bangalore. From each city, we will select two treatment centers and two delivery centers (hospitals). The cases and controls will be recruited over a period of 6 months from each site.

Any child with cleft lip with or without cleft palate or only cleft palate visiting the hospital for treatment (treatment center) within 4 months of birth will be recruited in the study. Clefts may occur in the lip, the roof of the mouth (hard palate), or the tissue at the back of the mouth (soft palate). The mother of this child will be considered as a case. Cases will be recruited only if they belong to the catchment area of the hospital. We will define the catchment area of the hospital by reviewing the statistics of the previous years. Controls will be matched based on the parity of the mother (primigravida or multigravida).

Controls will be recruited from the maternity hospitals (delivery centers) nearby to the treatment centers with a delivery load of 10,000 deliveries. Also, district level tertiary hospitals (government) will be considered for recruitment of controls. Controls will be recruited and interviewed within 48 hours of the birth of the baby.

A researcher will be stationed round the clock at the treatment and recruitment centers in all the three cities. They will recruit a case only after the physician has confirmed the diagnosis.

**Inclusion and exclusion criteria for the selection of cases.**

Inclusion criteria

- Mothers of babies born with nonsyndromic OFCs
- Mothers of babies visiting the treatment center within 4 months of birth of baby and from the center’s catchment area

Exclusion criteria

- Not belonging to treatment center’s catchment area
- Syndromic cases of OFCs
- Mothers who are currently a part of another ongoing research study receiving any intervention

**Inclusion and exclusion criteria for the selection of controls.**

Inclusion criteria

- Mother of babies born at the delivery center and from the same geographical location
- Mother of a live birth without any birth defect

Exclusion criteria

- Mothers who are currently a part of any other ongoing research study receiving any intervention
- Any maternal complication that requires immediate emergency care

#### Study Tool

A semistructured questionnaire will be administered to cases and controls by our research team. This will gather details on sociodemographic aspects and details of reproductive, medical, and occupational history.

The primary exposure for the study is folic acid consumption in the periconceptional period in a mother. History of intake of folic acid tablets during the periconceptional period and dietary history would help elicit the exposure status. Other exposures likely to be associated with OFCs, such as intake of drugs during pregnancy, sex selection practices, and exposure to pesticides would be elicited from history.

#### Statistical Analysis

A database will be developed in MS Access. Data analysis will be done using STATA 14.0. Descriptive and inferential statistics output will be generated. The prevalence of exposure will be compared between cases and controls using logistic regression, adjusting for potential confounder effects as appropriate.

## Results

This research was supported by a Wellcome Trust Capacity Strengthening Strategic Award to the Public Health Foundation of India and a consortium of United Kingdom universities. The results from the individual studies will be available between 2016 and 2017.

## Discussion

### Trial Implications

This paper outlines the rationale and design of a multistage study including a systematic review determining the current trends in birth prevalence of OFCs and NTDs across India, followed by a cross-sectional, population-based study to determine prevalence of specified birth defects and deficiency of folic acid and vitamin B12 in the community and by a case control study to study association of folic acid deficiency with OFCs. The outcomes from this study will help inform the research community about the feasibility of involving grass root level functionaries for screening cases of OFCs and NTDs.

We anticipate certain challenges in conducting this study. Identification of some of these is likely to be an intermediate outcome of this study as well as help in addressing the challenges involved in implementation of national programmes for early identification and early intervention.

### Challenges

Birth defects being considered a social taboo in India, we may have difficulty in getting mothers to participate in the study. Also, the prevalence of some of the specified birth defects is low and it may be difficult to identify these children in the community. For the case control study, it may be very difficult to get cooperation from the cases, as they may want to tend to the child. The deliverables and the outcomes from this research will drive strategies in minimizing the adverse public health consequences of folic acid deficiency among mothers in India and strengthen the evidence base in childhood disability for planning and policy initiatives.

## Abbreviations

ANM: auxiliary nurse midwives
FGD: focus group discussion
IDI: in-depth interview
KI: key informant
KIM: Key informant method
NIN: National institute of Nutrition
NTDs: neural tube defects
OFCs: orofacial clefts
